# Plastome phylogenomics and phylogenetic diversity of endangered and threatened grassland species (Poaceae) in a North American tallgrass prairie

**DOI:** 10.1002/ece3.6484

**Published:** 2020-06-25

**Authors:** Phyllis H. Pischl, Sean V. Burke, Elizabeth M. Bach, Melvin R. Duvall

**Affiliations:** ^1^ Department of Biological Sciences Northern Illinois University DeKalb Illinois USA; ^2^ Center for Translational Data Science University of Chicago Chicago Illinois USA; ^3^ The Nature Conservancy, Nachusa Grasslands Franklin Grove Illinois USA

**Keywords:** chloroplast, endangered species, phylogenetic diversity, phylogenomics, plastid, plastomes, Poaceae

## Abstract

Native grasslands are one of the most endangered ecosystems in North America. In this study, we examined the ecological and evolutionary roles of endangered and threatened (e/t) grasses by establishing robust evolutionary relationships with other nonthreatened native and introduced grass species of the community. We hypothesized that the phylogenomic distribution of e/t species of grasses in Illinois would be phylogenetically clustered because closely related species would be vulnerable to the same threats and have similar requirements for survival. This study presents the first time a phylogeny based on complete plastome DNA of Poaceae was analyzed by phylogenetic diversity analysis. To avoid the disturbance of e/t populations, DNA was extracted from herbarium specimens. Next‐generation sequencing (NGS) techniques were used to sequence DNA of plastid genomes (plastomes). The resulting phylogenomic tree was analyzed by phylogenetic diversity metrics. The extracted DNA successfully produced complete plastomes demonstrating that herbarium material is a practical source of DNA for genomic studies. The phylogenomic tree was strongly supported and defined *Dichanthelium* as a separate clade from *Panicum*. The phylogenetic metrics revealed phylogenetic clustering of e/t species, confirming our hypothesis.

## Graphical abstract

In this study, we examined the ecological and evolutionary roles of endangered and threatened grasses by establishing robust evolutionary relationships with other nonthreatened native and introduced grass species of the community. We present a modified technique to extract DNA from herbarium specimens and use phylogenetic analysis to reveal phylogenetic clustering of the endangered and threatened species.

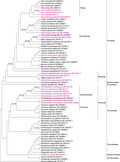

## 1 INTRODUCTION

Ecologists and conservation biologists are increasingly using phylogenetic diversity to better understand the connection between evolutionary history and ecological similarities in species assemblages. Phylogenetic diversity (PD) is a metric that measures the evolutionary distance between species in an assemblage (Faith, 1992). The use of phylogenetic diversity metrics may help conservation biologists preserve biodiversity in multiple ways. Understanding the phylogenetic diversity of a plant community may help conservation biologists identify plant communities with vital ecosystem functions. Plant communities with higher phylogenetic diversity have been shown to be more productive, and resistant to invasion (Barak et al., 2017). Phylogenetic diversity may also reveal plants at risk because they demonstrate phylogenetic clustering (Larkin et al., 2015). These species may be more closely related than expected by chance and share traits that make them vulnerable to the same threats. Such species are at a higher risk of extirpation due to habitat loss or changes in environmental conditions (Larkin et al., 2015; Liu, Edwards, Freckleton, & Osborne, 2012). In contrast, assemblages that are less phylogenetically related than predicted by chance provide evidence of phylogenetic overdispersion, potentially resulting from competitive exclusion. In some systems, including the tallgrass prairies of North America, phylogenetic diversity has been found to be a better predictor of ecosystem function than species richness or functional diversity (Barak et al., 2017; Barber et al., 2017; Wiens et al., 2010).

The tallgrass prairie is one of the most endangered ecosystems in North America (Henwood, 2010) and is largely being lost to anthropogenic conversion to agricultural lands (Lauenroth, Burke, & Gutmann, 1999). Since 1,830 when European settlement began, declines of 82%–99% of the native prairie have occurred across the North American grassland region (Samson & Knopf, 1994). As recently as 2006–2011, almost 530,000 ha of grassland was converted for agricultural purposes (Kane et al., 2017). The extensive loss of tallgrass prairie habitat has pushed many species to become endangered or threatened (e/t), including the defining family of the system: Poaceae (grasses). Poaceae consists of over 12,000 grass species in 12 subfamilies divided into 771 genera (Soreng et al., 2015).

Although all species in Poaceae are members of the same family, there is considerable divergence within this lineage. Tracing the PD of Poaceae species within tallgrass prairie, including endangered species as well as non‐native species, to the subfamily and tribal levels, provided insights into the evolution of these individuals and the niches they fill in their environment. Grasses provide numerous ecosystem services including erosion control, soil formation, habitat for wildlife (Costanza et al., 1997), and carbon storage (Samson & Knopf, 1994). Species of Poaceae are able to occupy diverse habitats due to the two photosynthetic pathways, C_3_, which is more common among grasses of cool regions, and C_4_, which has an advantage for species in warm and dry areas (Judd, Campbell, Kellogg, Stevens, & Donoghue, 2008). Members of Poaceae also express a variety of traits that give competitive advantages in disturbance‐dependent ecosystems, like the tallgrass prairie. For example, lateral shoots that are protected belowground allow some members of Poaceae to rapidly regrow after grazing or fire. The results of these investigations demonstrated the degree to which evolutionary history can identify unique and irreplaceable qualities of tallgrass prairie grasses as well as threats from current and potential non‐native species of grasses.

Illinois is nicknamed the “Prairie State” because tallgrass prairies once covered it. However, 99.9% of Illinois’ original tallgrass prairie was lost to the encroachment of agriculture, industry, and urbanization (Ellis, 2017). Of the over 300 species of Poaceae that are found in Illinois, there are seventeen endangered and one threatened species that are listed on the *Checklist Of Illinois Endangered And Threatened Animals And Plants* (IESPB, 2015). Many of these species of grasses are listed by Illinois as e/t species are also species of concern in neighboring states, either endangered, threatened, or rare. Despite being ecologically significant, including several e/t species, phylogenetic relationships within Poaceae of the tallgrass prairie are not well understood. Lack of complete genetic information from these species is a key hurdle to building highly supported phylogenies that can yield new evolutionary and ecological insights.

Including more informative DNA characters in phylogenetic analyses leads to phylogenies with greater support than studies using only gene coding sequences (e.g., Burke, Ungerer, & Duvall, 2018; Burke, Wysocki, et al., 2016). For Poaceae species, the ability to assemble complete plastid genomes (plastomes) allows for deep molecular sampling of both coding and noncoding sequences, the latter of which are less subject to the pressures of selection than genes. These noncoding sequences often carry historical data not seen in coding sequences and reveal important information for determining the evolutionary relationships between species (Saarela et al., 2018). This is the first phylogenetic study of complete plastomes from Poaceae that are used for phylogenetic diversity analyses.

In this study, we inferred evolutionary trees from complete plastomes of 68 species of grasses found to grow in Illinois (Taft, Wilhelm, & Ladd, 1997). These grassland species include three groups: the 18 e/t species, 14 native species, and 36 introduced species. We hypothesize that the phylogenomic distribution of e/t species of grasses in Illinois will be phylogenetically clustered because closely related species would be vulnerable to the same threats and have similar requirements for survival. This study informs ecologists of not only the biodiversity of Poaceae, but also the genetic diversity they represent. Because funding for conservation efforts is often limited, the genetic information provided by this research can help prioritize species or ecosystems for conservation as well as providing insight for other systematic and evolutionary studies.

## 2 METHODS

### 2.1 Species of grasses sequenced for this study

For the selection of e/t species of grasses, we followed the *Checklist Of Illinois Endangered And Threatened Animals And Plants* (IESPB, 2015), which lists seventeen endangered and one threatened species of Poaceae. Of the eighteen e/t species, the plastome for only four species had been sequenced and published prior to this work (Duvall et al., 2016; Saarela et al., 2015). The remaining fourteen e/t species, one duplicate specimen of *Dichanthelium commutatum* (Schult.) Gould, two species closely related to the e/t species, *Panicum dichotomum* L. and *Glyceria septentrionalis* (Fernald) Steyerm. & C.L.Kucera, and a native Illinois species, *Danthonia spicata* (L.) Roem. & Schult., comprised the eighteen species sequenced in this investigation. Appendix S1 records the Illinois e/t species as listed by the state of Illinois and the taxonomy for the specimens used in this study. The determination of which species of grasses are native or introduced to Illinois was according to Taft et al. (1997).

### 2.2 DNA extraction

In this study, we present 18 newly sequenced complete plastomes. Silica dried leaf material was used for *Beckmannia syzigachne* (Steud.) Fernald, *Calamagrostis pickeringii* (Swallen) C.W. Greene, and *Danthonia spicata*, and DNA was extracted according to manufacturer's protocol using DNeasy Plant Mini Kit (Qiagen). Leaf material for the remaining 15 plastomes was obtained with permission from preserved herbarium specimens (Table 1). The DNA extraction procedure was modified to recover DNA from these, sometimes decades‐old, herbarium specimens from known localities. This avoided further disturbance of living populations and eliminated the need for permits to collect from live, e/t plants. The leaf tissue was homogenized in liquid nitrogen, and DNA was extracted using the DNeasy Plant Mini Kit (Qiagen). Modifications to the manufacturer's protocol included increasing the starting material, extending the cell lysis incubation to 30 min, and increasing the 5‐min incubation on ice to seven days at −20°C similar to suggestions in Drábková, Kirschner, and Vlĉek (2002).

**TABLE 1:** Vouchers, accession numbers, and lengths of subregions for newly assembled plastomes

Taxon	Voucher/seed source	GenBank accession	Total plastome length (bp)	LSC (bp)	SSC (bp)	IR (bp)
*Beckmannia syzigachne* (Steud.) Fernald	PI 664238 USDA‐GRIN	MN996272	136,236	80,350	12,852	21,517
*Calamagrostis pickeringii* (Swallen) C.W. Greene	Peterson & Saarela 20857 (CAN)	MN937348	136,682	80,660	12,688	21,667
*Danthonia spicata* (L.) Roem. & Schult.	DiGiovanni s.n. (DEK)	MN955297	134,245	79,706	12,331	21,104
*Deschampsia flexuosa* (L.) Trin.	Hill 29437 (ILLS)	MN944893	135,901	79,957	12,876	21,534
*Dichanthelium boreale* (Nash) Freckmann	Phillipe & Ebinger 41703 (ILLS)	MN955295	140,089	82,099	12,572	22,709
*Dichanthelium commutatum* (Schult.) Gould (Illinois)	Ebinger 14302 (EIU)	MN955296	139,591	81,602	12,577	22,706
*Dichanthelium commutatum* (Schult.) Gould (Florida)	Sørensen 02‐06 (DEK)	MN970099	139,577	81,588	12,577	22,706
*Dichanthelium dichotomum* (L.) Gould (Illinois)	Edgin 3032 (EIU)	MN970100	140,105	82,104	12,575	22,713
*Dichanthelium portoricense* (Desv. ex Ham.) B.F.Hansen & Wunderlin	Brunton & McIntosh (MICH)	MN970101	140,107	82,148	12,565	22,697
*Dichanthelium ravenelii* Scribn. & Merr.	Taylor 13086 (ILL)	MN983108	140,063	82,093	12,572	22,699
*Elymus trachycaulus* (Link) Gould ex Shinners	Friberg 1407 (DEK)	MN983109	135,060	80,665	12,769	20,813
*Glyceria septentrionalis* (Fernald) Steyerm. & C.L.Kucera	Keibler 3 (DEK)	MN983110	136,159	81,125	12,638	21,198
*Glyceria septentrionalis* var. *arkansana* (Fernald) Steyerm. & C.L.Kucera	Hill & Koontz 33782 (ILLS)	MN983111	136,143	81,106	12,657	21,190
*Panicum dichotomum* L. (Indiana)	Crane 99‐462 (ILLS)	MN983112	140,151	82,155	12,570	22,713
*Poa alsodes* A. Gray	Spyreas et al. 192 (ILLS)	MN983113	136,070	80,168	12,832	21,535
*Poa saltuensis* Fernald & Wiegand	Taft & Solecki 780 (ILLS)	MN983114	136,140	80,285	12,833	21,511
*Poa wolfii* Scribn.	Hill & Traeger 30229 (ILLS)	MN983115	135,921	80,052	12,817	21,526
*Schizachne purpurascens* (Torr.) Swallen	Handel 1089 (ILLS)	MN983116	135,671	80,758	12,815	21,049
Average lengths (bp)			137,440	81,035	12,673	21,866

Abbreviations: GRIN, Germplasm Resources Information Network (https://www.ars‐grin.gov); IR, Inverted Repeat; LSC, Large Single Copy; SSC, Small Single Copy.

### 2.3 Library preparation and sequencing

The plastomes of the e/t species of grasses were processed following procedures similar to those used by Burke, Wysocki, et al. (2016). DNA libraries were prepared with the Nextera sample preparation protocol following manufacturer's instructions (Illumina). DNA samples were diluted to 2.5 ng/μl (50 ng total), and single‐end libraries were prepared. The libraries were sequenced as single‐pass runs on an Illumina HiSeq 3500 instrument at the Core DNA facility at Iowa State University (Ames). The only exception was *Beckmannia syzigachne*, which was prepared as a library with the Nextera XT sample preparation protocol following manufacturer's instructions, and DNA samples were diluted to 0.2 ng/μl (1 ng total). *Beckmannia syzigachne* and *Calamagrostis pickeringii* were sequenced on an Illumina HiSeq 2500 system.

### 2.4 Plastome assembly and verification

The plastomes of the DNA samples were assembled following procedures used by Burke, Wysocki, et al. (2016). The Illumina reads of low quality were removed at default settings (DynamicTrim, SolexaQA++, Cox, Peterson, & Biggs, 2010), remaining adapters attached to the reads were excised (CutAdapt, Martin, 2011), and all reads <25 base pairs (bp) in length were discarded (LengthSort, SolexaQA++, Cox et al., 2010). De novo assembly was performed using SPAdes v3.6.1 (Bankevich et al., 2012) with *k*‐mers set from 19 to 73 bp with intervals of six. An exception was the plastome for *Calamagrostis pickeringii*, in which de novo assembly was performed using Velvet version 1.2.08 (Zerbino & Birney, 2008) with *k*‐mers set from 19 to 85 bp with intervals of six. Redundant sequences were removed from the contig file (CD‐Hit v4.6, Fu, Niu, Zhu, Wu, & Li, 2012). Contigs were scaffolded using ACRE (Wysocki et al., 2014).

The ACRE scaffolds and clean reads were imported into Geneious 10.2.4 (Biomatters; Kearse et al., 2012). For each new species, a closely related plastome banked at NCBI was chosen as a reference. Using the MAFFT v7.308 (Katoh & Standley, 2013) plug‐in in Geneious, the contigs were aligned to the reference. Gaps between the contigs were closed by in silico genome walking using the “map to reference” function in Geneious. For each new plastome, a final verification was performed by mapping the reads to their respective complete assembly and manually adjusting any incongruences. Mean read depth was determined for each new complete plastome.

### 2.5 Plastome annotation

Each newly verified plastome was pairwise aligned with the closely related reference plastome, and annotations were transferred to the new plastome using the “transfer annotation” feature in Geneious. The gene coding sequences were examined, and boundaries were manually adjusted to preserve the correct reading frame. Following the method of Burke, Grennan, and Duvall (2012), inverted repeat (IR) boundaries were located. Using BLASTn (Altschul et al., 1997) to align the new plastome sequence to itself, segments in which the orientation of the reads transitioned from plus/plus to plus/minus indicated the IR boundaries. These segments were then located in the plastome sequences using the motif feature in Geneious, and IR annotations were added.

### 2.6 Phylogenomic analyses

Two plastome DNA matrices were assembled. The first matrix contained a total of 72 Poaceae species, including all 18 newly sequenced complete plastomes, four previously published e/t species, 49 Poaceae species found in Illinois (Taft et al., 1997), and the out‐group species, *Pharus latifolius* L. (Appendix S2). Any basal grade species of Poaceae could serve as an out‐group for the in‐group crown grasses analyzed here. Of the three basal lineages (Anomochlooideae, Puelioideae, and Pharoideae), only Pharoideae extends into the United States and so has a native range that is geographically closest to Illinois among the candidate out‐groups (http://www.emonocot.org/). Previously published complete plastomes were downloaded from NCBI (Geer et al., 2009). This matrix contained approximately 1.9 million newly sequenced base pairs. The second matrix contained a total of 69 Poaceae species including all 18 e/t species, 50 Poaceae species known to grow in Illinois (Taft et al., 1997), and the out‐group. The duplicate e/t specimen for *Dichanthelium commutatum*, the synonymous *Panicum dichotomum*, and the closely related specimen *Glyceria septentrionalis* were removed from the matrix to avoid redundancy in the phylogenetic diversity analysis. Both matrices were analyzed by the same methods. First, an alignment of the complete plastomes, excluding one copy of the IR, was generated in Geneious with MAFFT v7.308 (Katoh & Standley, 2013) plug‐in using the default settings. Then, all gaps that were produced by the alignment in one or more sequences were excluded from the matrix to eliminate the most ambiguously aligned regions (Burke, Lin, Wysocki, Clark, & Duvall, 2016) using the “stripping” feature of Geneious.

Using jModelTest v2.1.10 (Darriba, Taboada, Doallo, & Posada, 2012), the GTR + I + G model was selected under the Akaike information criterion (Akaike, 1974) for the gap‐free nucleotide alignments. Maximum‐likelihood (ML) and Bayesian MC3 inference (BI) analyses were conducted on the data sets. The ML analyses were performed using the RAxML‐HPC2 on XSEDE v8.2.10 (Stamatakis, 2014) at the CIPRES Science Gateway (Miller, Pfeiffer, & Schwartz, 2010). The number of bootstrap replicates was set to 1,000, and all model parameters were set to default values. The BI analyses were performed using MrBayes on XSEDE v3.2.6 (Ronquist et al., 2012) at the CIPRES Science Gateway. Each MrBayes analysis was set for two independent runs with four chains and twenty million generations each with a default burn‐in value of 25%. The substitution model was set to “invgamma” and “nst = 6,” and all other parameters were set at default settings.

### 2.7 Pairwise analysis of closely related specimens

Four pairs of plastomes were compared to each other. Two newly sequenced plastomes for *Dichanthelium commutatum* from specimens that were collected from different places at different times were compared. One specimen was collected in Illinois in 1973, and the other was collected in Florida in 2002. A pair of newly sequenced plastomes under the synonyms, *Dichanthelium dichotomum* (L.) Gould and *Panicum dichotomum*, were compared. Both were collected in 1999 in the neighboring Midwestern states. A newly sequenced plastome for the species *Glyceria septentrionalis* was compared to that from *Glyceria septentrionalis* var. *arkansana* (Fernald) Steyerm. & C. L. Kucera. These specimens were collected in the same state ten years apart, 1991 and 2001, respectively. *Dichanthelium commutatum* (2002) and *Dichanthelium dichotomum* were compared as example of congeneric species. For each pair, an alignment of the complete plastomes was generated using the “pairwise alignment” feature in Geneious with the MAFFT v7.308 (Katoh & Standley, 2013) plug‐in using the default settings.

### 2.8 Phylogenetic diversity analyses

Three phylogenetic diversity (PD) metrics were estimated in R (R Core Team, 2017) using the picante package (Kembel et al., 2010). To remove the effect of species richness (the number of taxa in each assemblage), the standardized effect sizes of phylogenetic diversity (SES.PD), mean pairwise distance (SES.MPD), and mean nearest taxon distance (SES.MNTD) were used. These metrics evaluated the phylogenetic evenness or clustering of the species in the assemblages tested (Kellar, Ahrendsen, Aust, Jones, & Pires, 2015). Mean pairwise distance measures the average of the evolutionary distance between all pairs of species in the assemblage, and mean nearest taxon distance measures the average of the evolutionary distance between each species and its nearest relative (Barber et al., 2017). Standardized effect size metrics are calculated by comparing the observed value to a randomly generated data set (Kellar et al., 2015). There were 10,000 randomizations in each standardized effect size analysis. The PD metrics were calculated for seven community assemblages of the 69 species phylogenetic matrix. Specimens were categorized as endangered/threatened species (E/T), native species not E/T (Nat), or introduced species (Intro) (Taft et al., 1997), and seven combinations of these categories produced the seven species assemblages. Subsequent designations of these assemblages were abbreviated as follows: “AIP” (all 68 Illinois Poaceae), “Intro + Nat,” “Nat + E/T,” “Intro + E/T,” “Nat,” “Intro,” and “E/T.”

## 3 RESULTS

### 3.1 Newly sequenced plastomes

The eighteen newly assembled complete plastomes were submitted to GenBank, and accession numbers were obtained (Table 1). All the newly sequenced plastomes exhibited the quadripartite structure with the same gene content and gene order that is typical of grasses (Burke, Wysocki, et al., 2016). The average length of the new plastomes was 137,440 bp, and they ranged in length from 134,245 to 140,151 bp. The number of reads for the data files for the new species averaged 16,475,004 reads that created an average read depth of 115×. These reads were assembled into an average of eight contigs after de novo assembly (Table 2).

**TABLE 2:** Next‐generation sequencing details for newly assembled Poaceae plastomes from this study

Taxon	Number of reads	Mean coverage	Number of scaffolded contigs
*Beckmannia syzigachne* (Steud.) Fernald	1,158,013	45.9	5
*Calamagrostis pickeringii* (Swallen) C. W. Greene	14,243,269	30.1	7
*Danthonia spicata* (L.) Roem. & Schult.	26,455,430	105	11
*Deschampsia flexuosa* (L.) Trin.	21,364,913	64.4	7
*Dichanthelium boreale* (Nash) Freckmann	19,930,267	277.5	14
*Dichanthelium commutatum* (Schult.) Gould (Illinois)	17,734,971	28.9	6
*Dichanthelium commutatum* (Schult.) Gould (Florida)	25,200,152	111.1	11
*Dichanthelium dichotomum* (L.) Gould (Illinois)	17,475,116	49.5	11
*Dichanthelium portoricense* (Desv. ex Ham.) B. F. Hansen & Wunderlin	15,759,828	55.0	13
*Dichanthelium ravenelii* Scribn. & Merr.	2,945,180	78.4	5
*Elymus trachycaulus* (Link) Gould ex Shinners	10,977,624	24.7	4
*Glyceria septentrionalis* (Fernald) Steyerm. & C. L. Kucera	25,256,562	84.6	3
*Glyceria septentrionalis* var. *arkansana* (Fernald) Steyerm. & C. L. Kucera	25,473,587	113.0	6
*Panicum dichotomum* L. (Indiana)	22,390,233	117.1	15
*Poa alsodes* A. Gray	12,370,167	161.0	7
*Poa saltuensis* Fernald & Wiegand	7,143,522	168.0	7
*Poa wolfii* Scribn.	11,645,076	178.0	5
*Schizachne purpurascens* (Torr.) Swallen	19,026,161	382.6	9
Averages	16,475,004	115	8

### 3.2 Phylogenomic analyses

**FIGURE 1:**
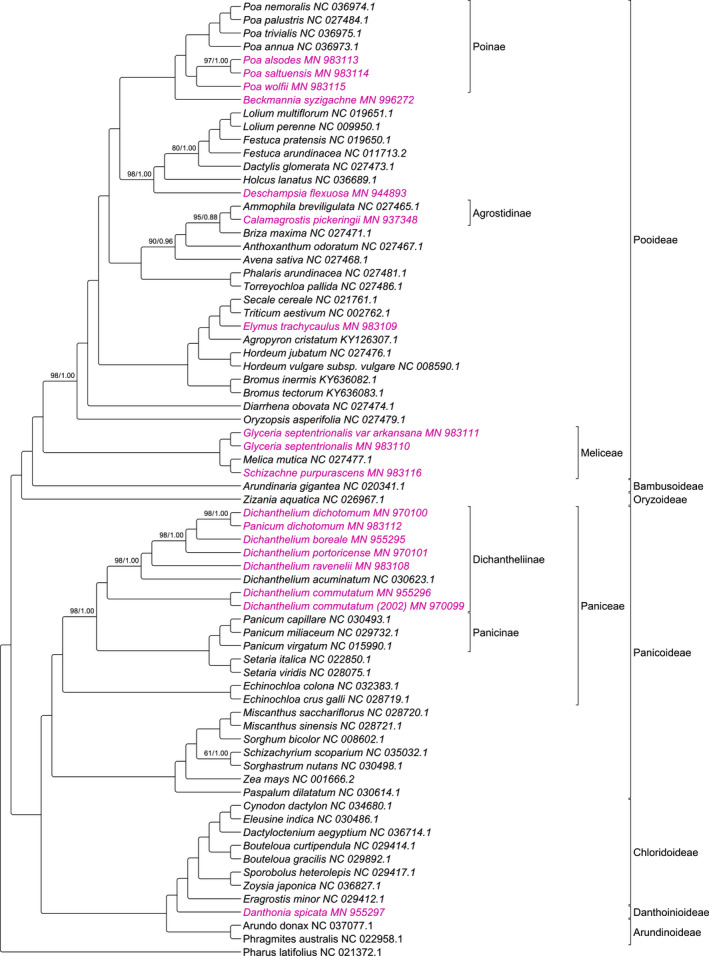
Phylogenomic tree of 72 Poaceae species. Newly sequenced plastomes are highlighted in purple. Nodes are maximally supported at bootstrap values (BV) of 100 and posterior probabilities (PP) of 1.0 except where noted (ML‐BV/BI‐PP)

After the removal of the second inverted repeat (IR) and all columns with gaps, the alignment of the 72 species containing all the newly sequenced plastomes included 86,658 nucleotide sites, 43,136 bp of which were variable, and the pairwise identity was 95.8%. The maximum‐likelihood (ML) and Bayesian MC3 inference (BI) analyses resulted in congruent topologies (Figure 1). Nodes were maximally supported except for 11 nodes in the ML analysis and two nodes in the BI analysis. The duplicate specimens for *Dichanthelium commutatum* were sister in the trees with maximum support. *Dichanthelium dichotomum* and the synonymously named *Panicum dichotomum* were also sister with a bootstrap value of 98 and posterior probability of 1.00. *Glyceria septentrionalis* var. *arkansana* and *Glyceria septentrionalis* were also sister taxa with maximal support.

**FIGURE 2:**
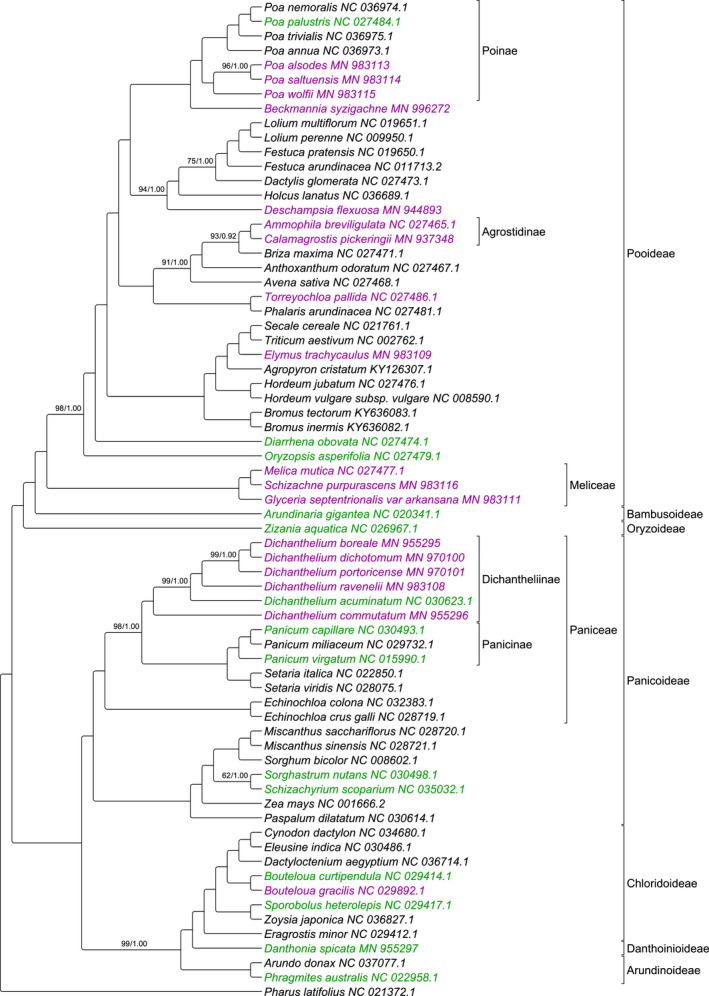
Phylogenomic tree of 69 Poaceae species. Endangered/threatened species are highlighted in purple. Nonthreatened native species are highlighted in green. Nodes are maximally supported at bootstrap values (BV) of 100 and posterior probabilities (PP) of 1.0 except where noted (ML‐BV/BI‐PP)

The alignment of the 69 species contained 86,609 nucleotide sites after the removal of the second IR and all columns with gaps. Of these sites, 43,081 bp was variable, and the alignment had 95.7% pairwise identity. The ML and BI analyses resulted in congruent topologies (Figure 2). There was maximum support for nodes except for 11 nodes in the ML analysis and one node in the BI analysis.

The species sampling provided broad coverage of the family Poaceae with representatives from seven of the nine major subfamilies present. The *Dichanthelium* spp. form a monophyletic group separate from the *Panicum* spp. in both of the phylogenomic trees with the exception of *Panicum dichotomum* in the 72 grasses spp. tree.

### 3.3 Pairwise alignment of closely related species

The pairwise identity among the two newly sequenced plastomes for *Dichanthelium commutatum* was 99.9%. The alignment was 139,612 bp, of which 139,532 bp was identical. For the alternately named *Dichanthelium dichotomum* and *Panicum dichotomum*, the pairwise alignment had a length of 140,176 bp of which 140,051 bp was identical, resulting in a 99.9% pairwise identity. The congeneric species, *Dichanthelium commutatum* (2002) and *Dichanthelium dichotomum*, had 99.1% pairwise identity and an alignment length of 140,293 bp with 139,071 identical bp. Finally, the pairwise identity among *Glyceria septentrionalis* and *Glyceria septentrionalis* var. *arkansana* was 99.9% and an alignment length of 136,207 bp of which 136,049 bp was identical.

### 3.4 Phylogenetic diversity analyses

The values of the three metrics, phylogenetic diversity (PD), mean pairwise distance (MPD), and mean nearest taxon distance (MNTD) (Table 3) are correlated with the species richness (number of taxa) (Barber et al., 2017; Kellar et al., 2015). This trend is seen in the PD results except for two instances in which the PD result is lower for the larger group (18 species in E/T is less than 14 species in Nat and 54 species in Intro + E/T is less than 50 species in Intro + Nat, Table 3) when the standardized effect sizes of the phylogenetic diversity metric (SES.PD) are significantly clustered for the larger group. The standardized effect size (SES) metrics are not influenced by this correlation because it compares the metric in the data set to a null mean value from randomly generated data sets of the phylogeny. The SES.PD for AIP, Intro + E/T, and E/T demonstrated significant phylogenetic clustering with negative values for SES.PD and *p* values <.05 (Kembel et al., 2010), although removing the E/T species from the AIP community assemblage of phylogenetic tree (Nat + Intro) results in a positive value for SES.PD that would suggest phylogenetic evenness. This is not distinguishable from a random assemblage as the *p* value = .5347 (Table 3).

**TABLE 3:** Phylogenetic diversity metric results for seven community assemblages from the phylogenomic tree in Figure 2

Community assemblage	Number of species	Metric	Standardized effect size (SES) metric	*p* value (quantile)	Interpretation of SES metric
		PD	SES.PD		
AIP	68	0.4629	−4.7566	.0073	PC*
Intro + Nat	50	0.4028	0.1585	.5347	PO
Nat + E/T	32	0.2632	−1.5157	.0649	PC
Intro + E/T	54	0.3699	−2.5564	.0085	PC*
Nat	14	0.1859	0.3786	.6490	PO
Intro	36	0.2993	−0.9860	.1663	PC
E/T	18	0.1459	−2.9433	.0011	PC*
		**MPD**	**SES.MPD**		
AIP	68	0.0426	−3.5872	.0077	PC*
Intro + Nat	50	0.0441	1.2158	.8901	PO
Nat + E/T	32	0.0399	−2.3246	.0112	PC*
Intro + E/T	54	0.0423	−1.1732	.1239	PC
Nat	14	0.0385	−1.6255	.0533	PC*
Intro	36	0.0447	1.2629	.9001	PO
E/T	18	0.0372	−2.5064	.0084	PC*
		**MNTD**	**SES.MNTD**		
AIP	68	0.0077	−4.0036	.0074	PC*
Intro + Nat	50	0.0096	0.5564	.6956	PO
Nat + E/T	32	0.0091	−1.2934	.1014	PC
Intro + E/T	54	0.0072	−2.7208	.0067	PC*
Nat	14	0.0169	0.5533	.7121	PO
Intro	36	0.0092	−1.0318	.1533	PC
E/T	18	0.0070	−2.8269	.0009	PC*

Note

Specimen categories are all Illinois Poaceae (AIP), endangered/threatened species (E/T), native species not E/T (Nat), or introduced species (Intro).

Abbreviations: PC, phylogenetic clustering; PO, phylogenetic overdispersion or evenness.

* Significant interpretation. Each standardized effect size metric is the result of 10,000 randomizations.

The SES.MPD for the community assemblages AIP, Nat + E/T, Nat, and E/T resulted in values with negative results and *p* values <.05 indicating phylogenetic clustering (Kembel et al., 2010). Removal of the E/T species from the AIP community results in a positive value for SES.MPD but is indistinguishable from a random assemblage as the *p* value is not significant.

The SES.MNTD for the community assemblages AIP, Intro + E/T, and E/T resulted in negative values with significant *p* values indicating phylogenetic clustering (Kembel et al., 2010). Removal of the E/T species from the AIP community assemblage results in a positive value for SES.MNTD but not significantly different from a random assemblage.

## 4 DISCUSSION

### 4.1 Newly sequenced plastomes

This study demonstrates the use of herbarium specimens to obtain sequences of complete plastomes. Fifteen of the complete plastomes assembled were sequenced from herbarium specimen leaf tissue. Two of the oldest specimens were collected in 1973 making them 44 years old at the time of DNA extraction, and yet, sufficient DNA was recovered to construct a complete plastome. In some cases, the number of Illumina reads for species from herbarium specimens rivaled the number from fresh tissue (Burke, Wysocki, et al., 2016). Genome skimming technology has been variously used by plant scientists. Sensitivities of the methods to state of the source plant tissues (e.g., fresh vs. silica dried vs. herbarium material), differences in cytoplasmic/nuclear DNA ratios (in diploids vs. polyploids), and differing organelle density (as in C_3_ vs. C_4_ leaf architectures) yield variable and somewhat taxon‐specific recoveries. For example, Straub et al. (2012; a representative of an earlier plant plastome study), working with Apocynaceae, recovered 10.4% plastid DNA reads in their libraries. In our DNA libraries, often from polyploid species of grasses, only approximately 0.4% of the reads are from plastid DNA (S. V. Burke, unpublished observation). However, modern advances in assembly algorithms mean that high‐quality plastomes can still be assembled across a range of taxa and conditions, which now extends frequently, if not routinely, to herbarium material.

Other phylogenetic studies have used herbarium specimens to either sequence a limited number of genes (Silva et al., 2017) or complete plastomes for evolutionary research (Besnard et al., 2014). The modifications we made to the DNA extraction procedure increased the reliability of using herbarium specimens. The ability to extract DNA from herbarium specimens opens many new avenues for research of rare and endangered plants without the need to stress the few surviving individuals by taking tissue for analysis. The niche of an e/t species and its evolutionary relatedness among taxa in its community can be assessed using phylogenetic diversity metrics calculated from plastome phylogenies. The results of these analyses may help ecologists and conservation biologists prioritize which species and habitats are in greatest need of preservation. Also, extinct plants may be studied from herbarium specimens collected while they were still surviving, and their complete plastomes may be used to determine evolutionary history and proper taxonomy. The ability to use herbarium specimen tissue in conjunction with next‐generation sequencing techniques gives herbaria an important role in botanical science. The herbarium represents a wealth of information just waiting to be revealed.

### 4.2 Phylogenomic analyses

Evolutionary relationships in the phylogenomic trees were strongly supported as evidenced by the high posterior probabilities and bootstrap values. Using complete plastomes provided a robust analysis and confident inference of nodes in the trees (Burke, Wysocki, et al., 2016), due to the vast number of nucleotide sites in the alignments and the composition of the DNA sequences. Our use of complete plastome DNA included not only genes that are subject to the pressures of selection, but also noncoding sequences that are less subject to selection (Saarela et al., 2018). These sequences include historical data not seen in coding sequences and are therefore important in determining the evolutionary relationships between closely related species. Other studies have shown the importance of a clearly resolved phylogeny and that the increased quantity of phylogenetically informative sites are vital to calculating informative phylogenetic diversity metrics (Davies, Kraft, Salamin, & Wolkovich, 2012; Kellar et al., 2015).

In this study, we estimated the phylogenetic placement of e/t species of grasses among other grassland Poaceae. There are three groups of closely related e/t species in Pooideae (Figure 2): (a) a clade containing several *Poa* species along with *Beckmannia syzigachne*, (b) a clade in Agrostidinae containing *Ammophila breviligulata* Fernald and *Calamagrostis Pickeringii*, and (c) a clade of three species in Meliceae including *Melica mutica* Walter, *Schizachne purpurascens* (Torr.) Swallen, and *Glyceria septentrionalis* var. *arkansana*. Since there are only eight native species of *Poa* in Illinois, the loss of the three e/t *Poa* species would decrease native diversity by 37% in this genus. This is especially concerning because *Poa saltuensis* Fernald & Wiegand and *P. wolfii* Scribn. are the only native upland species of *Poa*, leaving the introduced species *P. arachnifera* Torr. as the only remaining upland species of *Poa* in the state if the other two were lost (Taft et al., 1997). Five e/t *Dichanthelium* species are grouped in Panicoideae. If these species were extirpated from the landscape, this would also cause a loss of genetic diversity in this genus.

The ecosystem services provided by these species would be lost if they should be extirpated from the state. For example, *A. breviligulata* plays an important role in stabilizing the sand dunes along Lake Michigan and collecting organic material to allow the colonization of other plant species (Hilty, 2012). *Dichanthelium dichotomum* serves as an important food source to sparrows and many other granivorous songbirds, as well as upland game birds and small mammals (Hilty, 2012). Understanding the roles and relationships these species of grasses maintain will give fundamental information to support future conservation efforts. This study will inform ecologists of not only the grassland biodiversity, but also the genetic diversity they represent.

### 4.3 Comparison of closely related taxa

Plastomes for *Dichanthelium dichotomum* and *Panicum dichotomum* have high sequence identities (99.9%) as would be expected from samples of the same species. These results are similar to those seen when the two plastomes for *Dichanthelium commutatum* were compared to one another with 99.9% identity. This similarity is in contrast to the comparison of two more distantly related congeneric species *Dichanthelium commutatum* and *D. dichotomum* which had 99.1% identity. *Dichanthelium dichotomum* and *Panicum dichotomum* are sister taxa (Figure 1) with the phylogram branch length of 1.4 × 10^–4^, ML bootstrap value of 98, and a posterior probability of 1.0. In the phylogenomic tree, the *Dichanthelium* species including “*Panicum” dichotomum* form a monophyletic group, supporting *P. dichotomum* as the same species as *D. dichotomum*. The segregation of a monophyletic *Dichanthelium* from *Panicum* in our phylogenomic trees confirms the work of Zuloaga, Salomón, and Scataglini (2015).

### 4.4 Phylogenetic diversity analyses

Aust, Ahrendsen, and Kellar (2015) and Ahrendsen, Aust, and Kellar (2016) found that the metrics used in this study, SES.PD, SES.MPD, and SES.MNTD, are good indicators of overall phylogenetic diversity of a community assemblage. Studies have found the original PD metric (Faith, 1992) to be strongly correlated with species richness (number of species) when there are <80 species in the species assemblage (e.g., Kellar et al., 2015; Tucker & Cadotte, 2013). However, our data did not follow this pattern for two species assemblages when the SES.PD was significantly clustered, and the larger assemblage had a lower PD value. In both cases, the larger assemblage contains the E/T species, suggesting that the E/T species are very strongly phylogenetically clustered.

The SES.PD value for the Intro + Nat species assemblage is not distinguishable from random (Table 3). This result provides a phylogenetically diverse platform on which to analyze the e/t species. When the e/t species are added to this assemblage, it becomes the AIP assemblage and the SES.PD result becomes significantly phylogenetically clustered. The clustering may be attributed to the e/t species as the SES.PD results for Intro + E/T and E/T species assemblages also demonstrate significant phylogenetic clustering.

The SES.MPD results are also indistinguishable from random for the Intro + Nat assemblage but are significantly clustered when the E/T species are added (AIP; Table 3). SES.MPD results for the Nat + E/T, Nat, and E/T assemblages are significantly phylogenetically clustered. The MPD metric quantifies the relatedness of species deeper in the branching pattern of the phylogeny (Barber et al., 2017; Kellar et al., 2015). This suggests that the native species, including the e/t species, may have shared traits because they remained closely related throughout their evolutionary history.

The SES.MNTD results for the Intro + Nat assemblage are not significantly different from random. However, when e/t species are added to the assemblage (AIP; Table 3), the SES.MNTD values indicate significant phylogenetic clustering. The MNTD metric is a measure of the phylogenetic distance to the nearest taxon for each species in the tree and relates to the clustering of terminal nodes instead of deep level clustering (Barber et al., 2017; Webb, Ackerly, McPeek, & Donoghue, 2002).

Taken together, these phylogenetic diversity metric values show that the e/t species are phylogenetically clustered at evolutionary points in both the past and more recently. Should these species be extirpated from the landscape, several small clades of native grass diversity would be lost. This loss would cut deep into the phylogenomic tree as well as removing many sister taxa near the branch tips.

Three possible explanations as to why these e/t species are so closely related include the following: (a) Introduced species are competing with the e/t species in a similar manner and reducing their abundance, (b) the e/t species have specific habitat needs such that destruction and loss of habitats are reducing populations, or (c) closely related species are demonstrating phylogenetic conservatism in the limited niche habitat available. These explanations are not mutually exclusive and may be working in combination for some e/t species.

Introduced species may compete with e/t species for nutrients and habitats. There are several invasive species of grasses in Illinois such as *Bromus tectorum* L. (Schachner, Mack, & Novak, 2008), *Microstegium vimineum* (Trin.) A. Camus (Gibson, Spyreas, & Benedict, 2002), *Bromus inermis* L.,* Schedonorus phoenix* (Scop.) Holub, and *Poa pratensis* L. (Schwartz & Gibson, 2014). These grasses have been shown to outperform native grasses (Schwartz & Gibson, 2014). Most invasive grasses are C_3_‐photosynthesizing and all the e/t species of grasses in this study, except for *Bouteloua gracilis* (Kunth) Lag. ex Griffiths, are also C_3_, which means that they would be competing for resources during the same growing seasons. Another invasive grass is *Agropyron cristatum* (L.) Gaertn. which was introduced in North America at the start of the 20th century to reseed abandoned agricultural areas (CABI, 2019) and is sister to the clade containing the e/t species *Elymus trachycaulus* (Link) Gould ex Shinners in the phylogenomic tree. Having such a closely related invasive species could be a threat to *E. trachycaulus*, which provides beneficial ecosystem services. *Elymus trachycaulus* is drought‐tolerant and able to survive high soil pH and has been used in restoration projects at Yellowstone and Glacier national parks (Thorne & Cardina, 2011). Another e/t species, *Bouteloua gracilis*, has been shown to be a possible competitor for *Agropyron cristatum* (Grant‐Hoffman, Clements, Lincoln, & Dollerschell, 2012). Thus, maintaining a healthy assemblage of native species could provide a more biodiverse community and continue ecosystem services.

The e/t species have specific habitat needs, such as sand dunes for *Ammophila breviligulata* and wetlands for *Torreyochloa pallida* (Torr.) Church which is an obligate wetland species (Milburn, Bourdaghs, & Husveth, 2007). The *Dichanthelium* species are mostly found in wooded areas (Gould & Clark, 1978) with varying requirements for moisture. These habitats are severely reduced in Illinois since the settlement of Europeans in the 1800s (Ellis, 2017) as they have been lost to human development and agriculture. The e/t species in this study are all listed as endangered or threatened because they have “restricted habitats or low populations” except for *Poa alsodes* A. Gray which is listed because it was once widespread but is now nearly extirpated from Illinois (Mankowski, 2012). Habitats where e/t species are found should be made a conservation priority to protect these native species and the genetic biodiversity they add to the plant community.

Our phylogenetic diversity metrics indicate that the e/t species in Illinois are significantly phylogenetically clustered. This, together with the shared ecological roles found within clades, is suggestive of phylogenetic niche conservatism. Phylogenetic niche conservatism occurs when closely related species are more ecologically similar than expected by Brownian motion descent with modification (Losos, 2008). According to other studies, this may be the effect of habitat filtering (Losos, 2008; Webb et al., 2002) in which closely related species are “filtered in” to a community (Wiens et al., 2010) where only ecologically similar species can exist in the environment. Other studies have found that poorly resolved phylogenies can inflate estimates of phylogenetic conservatism (Davies et al., 2012) and that increasing the number of informative characters in alignments leads to greater support for the relationships in the phylogeny and, thus, greater confidence in PD metric calculations (Kellar et al., 2015). Due to the use of complete plastomes, our phylogenies are fully resolved and well‐supported due to the large number of informative sites, increasing the reliability of our PD metrics. In their grassland studies, Ahrendsen et al. (2016), and Aust et al. (2015), included parallel analyses of single‐gene, dual‐gene, and multigene phylogenies. Their results also support our claims that more informative characters provide more robust support for relationships (Ahrendsen et al., 2016; Aust et al., 2015).

In a study of Cyperaceae, researchers were able to use phylogenetic diversity metrics, functional traits, and GIS to identify areas of high diversity as conservation priorities. Using phylogenetic histories, they were also able to reveal recently diversified endemic species, which may be globally unique (Spalink et al., 2018). Similar to our investigation finding phylogenetic niche conservatism in e/t species of grasses, these researchers found lineages of Cyperaceae that adapted to the Arctic biome that also exhibit phylogenetic niche conservatism (Spalink et al., 2018).

Over half of the e/t species in this study are only found in one or two counties in Illinois (Illinois Natural Heritage Database, 2016). Because our results show significant phylogenetic clustering, our PD metrics suggest that the e/t species occupy limited niches. Generally, the number of species per genus is lower in local areas (Webb et al., 2002), but a few genera of grasses are represented in Illinois native communities by several congeners, specifically in *Dichanthelium*,* Panicum*, and *Poa*. These observations and the evidence of phylogenetic clustering support our hypothesis that the e/t species are closely related to similar habitat needs and are vulnerable to the same threats. These findings reinforce the need for conservation of the niches occupied by these e/t species.

Further studies might explore phylogenetic diversity analyses of endangered and threatened species from other major families of grasslands, such as Asteraceae or Fabaceae, using complete plastome phylogenomics and phylogenetic diversity analyses similar to this study. Increasing the number of genes has been shown to give greater support for phylogenies (Kellar et al., 2015). Another study suggested that the inclusion of functional trait data can reveal the mechanisms of community assembly (Mason & Pavoine, 2013; Spalink et al., 2018). Continuing this line of study will indicate whether the mechanism that causes the e/t species of grasses to be phylogenetically clustered also operates across other major groups of the grassland plant community.

## 5 CONCLUSIONS

In this study, we have shown how herbarium material is useful for ecological research, allowing the study of e/t species without disturbing the few extant populations. DNA extracted from the herbarium material was used to produce complete plastome sequences using sequencing‐by‐synthesis techniques. The complete plastomes provided a robust and strongly supported phylogeny. Deep molecular sampling revealed information about the evolutionary relationships of species in the phylogeny. Communities of grasses in Illinois were evaluated using the standardized effect sizes of phylogenetic diversity metrics to remove the effect of species richness in the various subsets of the community assemblages. The three PD metrics all led to the same result; the e/t species are phylogenetically clustered, which can be interpreted as phylogenetic niche conservatism of these grasses.

The extirpation of the e/t grasses in this study would represent a loss of phylogenetic diversity deep in the Pooideae caused by the removal of the three Meliceae species and a loss of more recently diverged species in Agrostidinae and Poinae. Panicoideae would also lose most of the clade containing the members of *Dichanthelium*. The loss of the e/t species and the genetic biodiversity they supply would also lead to changes in ecosystem services and protection from invasive species (Lyons & Schwartz, 2001). The niches occupied by the e/t species should be considered as priority conservation sites to protect these species and the biodiversity and ecosystem services they provide. Maintaining healthy native plant communities is essential to other organisms that share their habitats and rely on these plants for shelter and forage, and to humans for the ecosystem services necessary for a healthy environment.

## CONFLICT OF INTEREST

None declared.

## AUTHOR CONTRIBUTION

**Phyllis H. Pischl:** Conceptualization (lead); Data curation (equal); Formal analysis (equal); Investigation (equal); Methodology (equal); Writing‐original draft (lead); Writing‐review & editing (equal). **Sean V. Burke:** Data curation (equal); Formal analysis (equal); Investigation (equal); Methodology (equal); Writing‐review & editing (equal). **Elizabeth M. Bach:** Conceptualization (supporting); Formal analysis (equal); Writing‐review & editing (equal). **Melvin R. Duvall:** Conceptualization (equal); Project administration (equal); Supervision (lead); Writing‐original draft (equal); Writing‐review & editing (equal).

## Supporting information

Appendix S1

Appendix S2

## Data Availability

DNA sequences: GenBank accessions MN937348, MN944893, MN955295–MN955297, MN970099–MN970101, MN983108–MN983116, and MN996272.
