# Supplementary material for: Plastome phylogenomics and phylogenetic diversity of endangered and threatened grassland species (Poaceae) in a North American tallgrass prairie
Source: Ecol Evol. 2020 Jun 25;10(14):7602–15. doi: 10.1002/ece3.6484 (PMC7391303; doi:10.1002/ece3.6484)
Supplement: Supplementary file 1 — Appendix S1 [file ECE3-10-7602-s001.docx]

**Appendix S1.** List of Endangered/Threatened Species taxonomy used in this study.

* indicates previously published plastome.

| **Endangered Species as listed (IESPB, 2015)** | **Alternate names according to theplantlist.org or plants.usda.gov** | **Species used in this study** |
| --- | --- | --- |
| *Beckmannia syzigachne* (Steud.) Fernald | *Beckmannia eruciformis* (L.) Host | *Beckmannia syzigachne* |
| *Bouteloua gracilis* [(Kunth) Lag. ex Griffiths](http://theplantlist.org/tpl1.1/record/kew-399440) | *Chondrosum gracile* Kunth  *Bouteloua oligostachya* (Nutt.) Torr. ex A.Gray | *Bouteloua gracilis** |
| *Calamagrostis insperata* Swallen | *Calamagrostis porteri* subsp.  *insperata* (Swallen) C.W.Greene | *Calamagrostis pickeringii* (Swallen) C.W.Greene  (congeneric species) |
| *Deschampsia flexuosa* (L.) Trin. | *Aira flexuosa* L. | *Deschampsia flexuosa* |
| *Dichanthelium bor*e*ale* (Nash) Freckmann | *Panicum boreale* Nash | *Dichanthelium boreale* |
| *Dichanthelium joori* (Vasey) Mohlenbr. | *Dichanthelium commutatum* (Schult.) Gould*Panicum commutatum* Schult. | *Dichanthlium commutatum* |
| *Dichanthelium portoricense* (Desv. ex Ham.) B.F.Hansen & Wunderlin | *Panicum portoricense* Desv. ex Ham. | *Dichanthelium portoricense* |
| *Dichanthelium ravenelii* (Scribn. & Merr.) Gould | *Panicum ravenelii* Scribn. & Merr. | *Dichanthelium ravenelii* |
| *Dichanthelium yadkinense* (Ashe) Mohlenbr. | *Dichanthelium dichotomum* (L.) Gould  *Panicum dichotomum* L. | *Dichanthelium dichotomum* |
| *Elymus trachycaulus* [(Link) Gould ex Shinners](http://theplantlist.org/tpl1.1/record/kew-411630) | *Agropyron trachycaulum* (Link) Steud.  *Elymus violaceus* (Hornem.) J.Feilberg | *Elymus trachycaulus* |
| *Glyceria arkansana* (Fernald) Steyerm. & C.L.Kucera | *Glyceria septentrionalis* var. *arkansana* (Fernald) Steyerm. & C.L.Kucera | *Glyceria septentrionalis* var. *arkansana* |
| *Melica mutica* Walter | Melica diffusa Pursh , Melica glabra Michx. | *Melica mutica** |
| *Poa alsodes* A. Gray | *Poa* *diantha* Steud. | *Poa alsodes* |
| *Poa languid* Hitchc. | *Poa saltuensis* Fernald & Wiegand | *Poa saltuensis* |
| *Poa wolfii* Scribn. | Poa alsodes *var.* wolfii (Scribn.) Scribn. | *Poa wolfii* |
| *Schizachne purpurascens* (Torr.) Swallen | *Avena torreyi* Nash*, Melica purpurascens* (Torr.) Hitchc. | *Schizachne purpurascens* |
| *Torreyochloa pallida* (Torr.) Church | *Glyceria fernaldii* (Hitchc.) H.St.John, *Glyceria pallid* (Torr.) Trin. | *Torreyochloa pallida** |
| **Threatened Species** |  |  |
| *Ammophila breviligulata* Fernald | Calamagrostis breviligulata (Fernald) Saarela | *Ammophila breviligulata** |
