# Supplementary material for: Plastome phylogenomics and phylogenetic diversity of endangered and threatened grassland species (Poaceae) in a North American tallgrass prairie
Source: Ecol Evol. 2020 Jun 25;10(14):7602–15. doi: 10.1002/ece3.6484 (PMC7391303; doi:10.1002/ece3.6484)
Supplement: Supplementary file 2 — Appendix S2 [file ECE3-10-7602-s002.docx]

**Appendix S2.** List of Poaceae species contained in the 72 species matrix according to e/t, native or introduced status. Newly sequenced plastome accession numbers in bold.

| Endangered/Threatened Species | |
| --- | --- |
| *Ammophila breviligulata* Fernald  NC_027465.1  *Beckmannia syzigachne* (Steud.) Fernald  **MN_996272**  *Bouteloua gracilis* (Kunth) Lag. ex Griffiths  NC_029892.1  *Calamagrostis pickeringii* (Swallen) C.W.Greene **MN_937348**  *Deschampsia flexuosa* (L.) Trin.  **MN_944893**  *Dichanthelium boreale* (Nash) Freckmann **MN_955295**  *Dichanthelium commutatum* (Schult.) Gould **MN_955296**  *Dichanthelium commutatum* (Schult.) Gould (2002) **MN_970099**  *Dichanthelium dichotomum* (L.) Gould **MN_970100** | *Dichanthelium portoricense* (Desv. ex Ham.) B.F.Hansen & Wunderlin **MN_970101**  *Dichanthelium ravenelii* Scribn. & Merr. **MN_983108**  *Elymus trachycaulus* (Link) Gould ex Shinners **MN_983109**  *Glyceria septentrionalis* var *arkansana* (Fernald) Steyerm. & C.L.Kucera **MN_983111**  *Melica mutica* Walter NC_027477.1  *Panicum dichotomum* L. **MN_983112**  *Poa alsodes* A. Gray **MN_983113**  *Poa saltuensis* Fernald & Wiegand **MN_983114**  *Poa wolfii* Scribn. **MN_983115**  *Schizachne purpurascens* (Torr.) Swallen **MN_983116**  *Torreyochloa pallida* (Torr.) Church  NC_027486.1 |
| Native Species | |
| *Arundinaria gigantea* (Walter) Muhl. NC_020341.1  *Bouteloua curtipendula* (Michx.) Torr. NC_029414.1  *Danthonia spicata* (L.) Roem. & Schult. **MN_955297**  *Diarrhena obovata* (Gleason) Brandenburg NC_027474.1  *Dichanthelium acuminatum* (Sw.) Gould & C.A.Clark NC_030623.1  *Glyceria septentrionalis* (Fernald) Steyerm. & C.L.Kucera **MN_983110** | *Oryzopsis asperifolia* Michx.  NC_027479.1  *Panicum capillare* L. NC_030493.1  *Panicum virgatum* L. NC_015990.1  *Phragmites australis* (Cav.) Trin. ex Steud. NC_022958.1  *Poa palustris* L. NC_027484.1  *Schizachyrium scoparium* (Michx.) Nash NC_035032.1  *Sorghastrum nutans* (L.) Nash NC_030498.1  *Sporobolus heterolepis* (Gray) A. Gray NC_029417.1  *Zizania aquatica* L. NC_026967.1 |
| Introduced Species | |
| *Agropyron cristatum* (L.) Gaertn. KY126307.1  *Anthoxanthum odoratum* L. NC_027467.1  *Arundo donax* L. NC_037077.1  *Avena sativa* L. NC_027468.1  *Briza maxima* L. NC_027471.1  *Bromus inermis* Leyss. KY636082.1  *Bromus tectorum* L. KY636083.1  *Cynodon dactylon* (L.) Pers. NC_034680.1  *Dactylis glomerata* L. NC_027473.1  *Dactyloctenium aegyptium* (L.) Willd. NC_036714.1  *Echinochloa colona* (L.) Link NC_032383.1  *Echinochloa crus-galli* (L.) P.Beauv. NC_028719.1  *Eleusine indica* (L.) Gaertn. NC_030486.1  *Eragrostis minor* Host NC_029412.1  *Festuca arundinacea* Schreb. NC_011713.2  *Festuca pratensis* Huds. NC_019650.1  *Holcus lanatus* L. NC_036689.1  *Hordeum jubatum* L. NC_027476.1  *Hordeum vulgare* subsp. *Vulgare* L. NC_008590.1 | *Lolium multiflorum* Lam. NC_019651.1  *Lolium perenne* L. NC_009950.1  *Miscanthus sacchariflorus* (Maxim.) Hack. NC_028720.1  *Miscanthus sinensis* Andersson NC_028721.1  *Panicum miliaceum* L. NC_029732.1  *Paspalum dilatatum* Poir. NC_030614.1  *Phalaris arundinacea* L. NC_027481.1  *Poa annua* L. NC_036973.1_Short_Single_Copy_Inverted  *Poa nemoralis* L. NC_036974.1  *Poa trivialis* L. NC_036975.1  *Secale cereal* L. NC_021761.1  *Setaria italic* (L.) P.Beauv. NC_022850.1  *Setaria viridis* (L.) P.Beauv. NC_028075.1  *Sorghum bicolor* (L.) Moench NC_008602.1  *Triticum aestivum* L. NC_002762.1  *Zea mays* L. NC_001666.2  *Zoysia japonica* Steud. NC_036827.1 |
| Outgroup Species | |
| *Pharus latifolius* L. NC_021372.1 | |
